# Nitrogen Flow in Diazotrophic Cyanobacterium *Aphanizomenon flos-aquae* Is Altered by Cyanophage Infection

**DOI:** 10.3389/fmicb.2020.02010

**Published:** 2020-08-19

**Authors:** Jolita Kuznecova, Sigitas Šulčius, Angela Vogts, Maren Voss, Klaus Jürgens, Eugenijus Šimoliūnas

**Affiliations:** ^1^Laboratory of Algology and Microbial Ecology, Nature Research Centre, Vilnius, Lithuania; ^2^Section Biological Oceanography, Leibniz Institute for Baltic Sea Research, Warnemünde, Germany; ^3^Department of Molecular Microbiology and Biotechnology, Life Sciences Center, Vilnius University, Vilnius, Lithuania

**Keywords:** ^15^N, *Aphanizomenon flos-aquae*, Baltic Sea, diazotrophy, nitrogen fixation, nanoSIMS, vB_AphaS-CL131, virus-host interactions

## Abstract

Viruses can significantly influence cyanobacteria population dynamics and activity, and through this the biogeochemical cycling of major nutrients. However, surprisingly little attention has been given to understand how viral infections alter the ability of diazotrophic cyanobacteria for atmospheric nitrogen fixation and its release to the environment. This study addressed the importance of cyanophages for net ^15^N_2_ assimilation rate, expression of nitrogenase reductase gene (*nifH*) and changes in nitrogen enrichment (^15^N/^14^N) in the diazotrophic cyanobacterium *Aphanizomenon flos-aquae* during infection by the cyanophage vB_AphaS-CL131. We found that while the growth of *A*. *flos-aquae* was inhibited by cyanophage addition (decreased from 0.02 h^–1^ to 0.002 h^–1^), there were no significant differences in nitrogen fixation rates (control: 22.7 × 10^–7^ nmol N heterocyte^–1^; infected: 23.9 × 10^–7^ nmol N heterocyte^–1^) and *nifH* expression level (control: 0.6–1.6 transcripts heterocyte^–1^; infected: 0.7–1.1 transcripts heterocyte^–1^) between the infected and control *A*. *flos-aquae* cultures. This implies that cyanophage genome replication and progeny production within the vegetative cells does not interfere with the N_2_ fixation reactions in the heterocytes of these cyanobacteria. However, higher ^15^N enrichment at the poles of heterocytes of the infected *A*. *flos-aquae*, revealed by NanoSIMS analysis indicates the accumulation of fixed nitrogen in response to cyanophage addition. This suggests reduced nitrogen transport to vegetative cells and the alterations in the flow of fixed nitrogen within the filaments. In addition, we found that cyanophage lysis resulted in a substantial release of ammonium into culture medium. Cyanophage infection seems to substantially redirect N flow from cyanobacterial biomass to the production of N storage compounds and N release.

## Introduction

Biological nitrogen (N_2_) fixation (conversion of dissolved N_2_ gas into ammonia by microorganisms) is an important process of the global biogeochemical cycles, considerably replenishing N losses to denitrification and anaerobic ammonium oxidation (Deutsch et al., 2007), and therefore sustaining carbon export and sequestration in the ecosystem (Karl et al., 2012). Nitrogen fixing microorganisms (diazotrophs) provide bioavailable nitrogen to the system in the form of ammonia and dissolved organic nitrogen that fuels primary and secondary production and, thus significantly contribute to the ecosystem functioning. Since cyanobacteria dominate epipelagic N_2_ fixation in both marine and freshwater environments, it is of great importance to understand factors controlling nitrogen fixation and release in these microorganisms (Benavides and Voss, 2015; Savchuk, 2018). It has been shown that both abiotic (e.g., nutrients, temperature, and mixing) and biotic (e.g., grazing) factors can influence diazotrophic activity and nitrogen distribution within the food web (reviewed in Benavides and Voss, 2015; Kuypers et al., 2018; Pajares and Ramos, 2019). However, the role of virus-host interactions in N_2_ assimilation in diazotrophic cyanobacteria and the effect of infection and lysis on the fate of fixed N_2_ is poorly described, representing a major knowledge gap in our understanding of the global nitrogen cycling.

It was suggested that viruses have the potential to impact nutrient cycling at scales from single cells to whole ecosystems (Brussaard et al., 2008; Shelford et al., 2012; Zimmerman et al., 2020). For example, it was shown that viral infection leads to the decreased carbon to nitrogen ratio (Ankrah et al., 2014), consequently resulting in stoichiometric imbalance of the infected cell and, thus, potential changes in its nutritional value. Recent studies of marine non-diazotrophic cyanobacteria *Synechococcus* demonstrated that viruses employ host nutrient uptake machinery to acquire extracellular nitrogen (in the form of nitrates) from the surrounding environment, which is then incorporated into newly synthesized viral particles (Pasulka et al., 2018; Waldbauer et al., 2019). In addition, viral genes encoding for proteins involved in nitrogen uptake (homolog of *amt*; Monier et al., 2017) or ammonium oxidation (homologs of *amoC* and *amoA*; Ahlgren et al., 2019) was found in viral metagenomes and culture isolates. Experiments demonstrated that these genes (e.g., *amt*) are expressed during infection and even enhance ammonium uptake rates by the infected cells (Monier et al., 2017). Moreover, as the infection proceeds viruses can switch from using host biomass-derived nitrogen to the extracellularly derived nitrogen sources to meet their N demand required for effective replication (Waldbauer et al., 2019). Lysis of the infected cells and release of dissolved organic and inorganic nitrogen (viral shunt; Wilhelm and Suttle, 1999) were shown to induce structural and functional changes in co-occurring microbial communities and promoted pelagic production due to increased remineralization of the released nutrients (Shelford et al., 2012). These examples suggest that viral infection and lysis can directly modulate N transformations in the ecosystems. From the perspective of atmospheric nitrogen dynamics in diazotrophic cyanobacteria, one thus could hypothesize that infection by viruses can induce alterations in (i) nitrogen assimilation (N_2_ fixation) and (ii) release (ammonium) rates, due to, for example, metabolic reprogramming of the host cells (Doron et al., 2016) as well as (iii) redistribution of N within the infected cells by redirecting intracellular nutrient pool toward production of new virions (Ankrah et al., 2014).

The *Aphanizomenon flos-aquae* is a bloom-forming heterocytous cyanobacterium, distributed worldwide in fresh and brackish water ecosystems (Cirés and Ballot, 2016). *A. flos-aquae* substantially contributes to the nitrogen pool in the Baltic Sea and fixes up to 75% of total fixed nitrogen in this ecosystem of which up to 50% are released (Ploug et al., 2010; Klawonn et al., 2016). *A. flos-aquae* and other filamentous cyanobacteria in the Baltic Sea fuel a microbial food web which is efficiently grazed by zooplankton in particular during the late phase of a bloom (Wannicke et al., 2013; Woodland et al., 2013; Karlson et al., 2015; Adam et al., 2016). Cyanophages were shown to significantly reduce population size of *A*. *flos*-*aquae* in laboratory incubations (Šulčius et al., 2015a). In addition, cyanophage infection can substantially alter the population structure of *A*. *flos-aquae* via reduction of the filament size and due to caused changes in ratio between vegetative cells, heterocytes and akinetes, since the latter two types of *A*. *flos*-*aquae* cells were shown to be insensitive to cyanophage additions (Šulčius et al., 2015a, 2017). Thus, this raises an intriguing question whether and to what extent cyanobacterial viruses (cyanophages) influences N_2_ fixation and nitrogen transformation processes in these cyanobacteria, and, in particular, whether viral infections alter the level of gene expression (e.g., *nifH*) and net incorporation of N_2_ into cyanobacterial biomass. In addition, there is a paucity of knowledge regarding how infection affects nitrogen flow within the filaments of the infected cyanobacteria. The lack of this information hampers our understanding of how virus-bacterium interactions influence biological nitrogen fixation process in the Baltic Sea and other aquatic ecosystems in which *A*. *flos-aquae* occurs at the high densities.

To advance our understanding of the effect of cyanophage infection on diazotrophic activity, we infected a culture of *A*. *flos-aquae* with the virulent cyanophage vB_AphaS-CL131 (hereafter CL 131; Šulčius et al., 2015a) in a short-term (36 h; within one infection cycle of CL 131) laboratory incubation experiment. We then followed changes in nitrogen fixation rates using a ^15^N_2_ stable isotope tracer addition (Montoya et al., 1996) and the transcript abundance of *nifH*, a marker gene for N_2_ fixation (Raymond et al., 2004), in infected *A*. *flos-aquae* cultures and compared these expression levels and fixation rates to those of uninfected controls. In addition, we used high-resolution nanometer-scale secondary ion mass spectrometry (nanoSIMS) of *A*. *flos-aquae* to evaluate the cellular distributions of ^15^N enrichments during infection as a proxy for nitrogen assimilation and flow among the cells within the filament.

## Results

### Dynamics of *A*. *flos-aquae* and Cyanophage vB_AphaS-CL131

**FIGURE 1:**
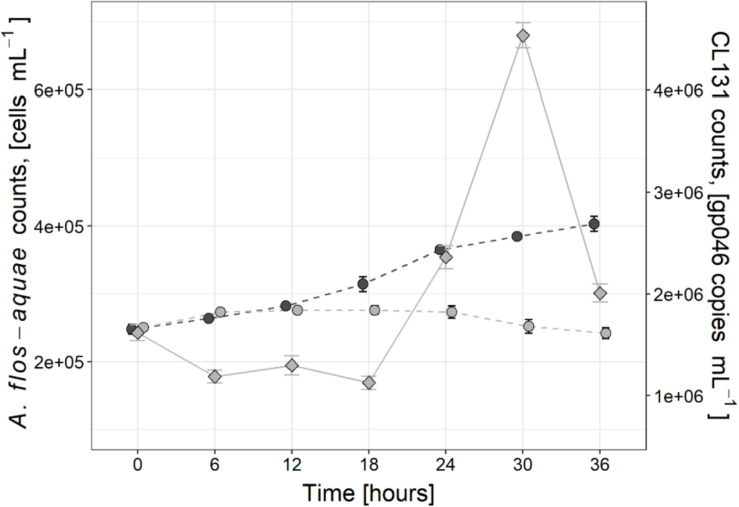
Changes in *Aphanizomenon flos-aquae* strain 2012/KM1/D3 cell abundance (circles) in the control (dark gray) and infected (light gray) treatments as well as dynamics of cyanophage vB_AphaS-CL131 gp046 gene copy numbers (diamonds) during the course of incubation experiment. Symbols indicate the mean values, and the error bar shows the standard deviation of three technical replicates.

In the control treatment without virus addition, the abundance of *A*. *flos-aquae* cells increased by 62.6% at the end of the experiment compared to its initial counts and changed from 2.5 × 10^5^ cells mL^–1^ (±0.7 × 10^3^ cells mL^–1^) to 4.0 × 10^5^ cells mL^–1^ (±1.1 × 10^4^ cells mL^–1^). No significant changes in *A*. *flos-aquae* abundance over the period of 36 h was observed in the infected culture (Figure 1), which indicated the suppression of *A*. *flos-aquae* population growth. The apparent growth rate of *A*. *flos-aquae* in the control culture was about 0.02 h^–1^ (±0.0008 h^–1^) and significantly (∼10-fold; *t*-test, *t* = 8.90, *p* < 0.0, *df* = 12) exceeded that of the infected culture [0.002 h^–1^ (±0.0009 h^–1^)].

Changes in cyanophage CL 131 abundances were assessed by enumerating gene copy numbers of the structural gene gp046 in cell-free filtrate (Figure 1). Six hours after cyanophage addition, the number of CL 131 cyanophages decreased to ∼73% of its initial numbers, indicating that about 1/4 of all cyanophages in the stock adsorbed to its host cells. The CL 131 density then significantly (Tukey HSD *p* < 0.00) increased 24 h after cyanophage addition (2.4 × 10^6^ gp046 gene copies mL^–1^ ± 9.2 × 10^4^ gp046 gene copies mL^–1^) and reached its peak in abundance at about 30 h post inoculation (4.5 × 10^6^ gp046 gene copies mL^–1^ ± 1.0 × 10^5^ gp046 gene copies mL^–1^; Figure 1).

### Nitrogen Fixation and Gene Expression

**FIGURE 2:**
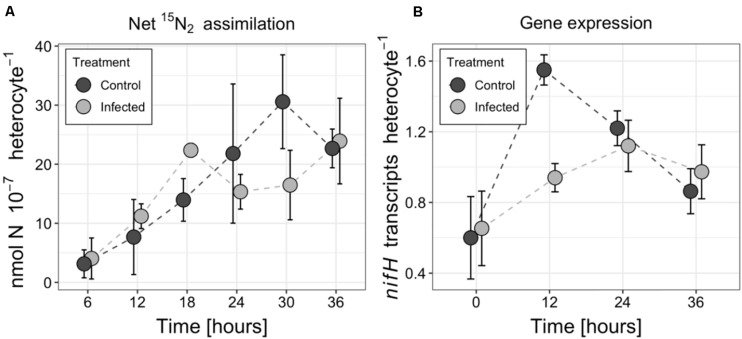
Changes in nitrogen fixation **(A)** and *nifH* transcript abundance **(B)** in the control and infected cultures of the *Aphanizomenon flos-aquae* strain 2012/KM1/D3. Symbols indicate the mean values, and the error bar shows the standard deviation of three technical replicates.

The net ^15^N_2_ fixation rates by *A*. *flos-aquae* cumulated over the course of incubation experiment in both treatments (Figure 2A), and eventually reached 22.7 × 10^–7^ nmol N heterocyte^–1^ (±3.3 × 10^–7^ nmol N heterocyte^–1^) and 23.9 × 10^–7^ nmol N heterocyte^–1^ (±7.3 × 10^–7^ nmol N heterocyte^–1^) in the control and infected culture, respectively. No statistically significant differences in N_2_ fixation rates were found between the infected and control treatments (RM ANOVA *F* = 2.25, *p* = 0.14, *df* = 1) over the course of the incubation experiment. These observations were consistent with changes in the number of *nifH* transcripts (Figure 2B), where no differences between the two treatments were detected (RM ANOVA *F* = 4.22, *p* = 0.06, *df* = 1), except for 12 h post inoculation. The number of *nifH* transcripts varied from 0.6 (±0.2) to 1.6 (±0.1) transcripts per heterocyte and from 0.7 (±0.2) to 1.1 (±0.2) transcripts per heterocyte in the control and infected cultures, respectively.

### Nitrogen Enrichments in *A*. *flos-aquae* Filaments

**FIGURE 3:**
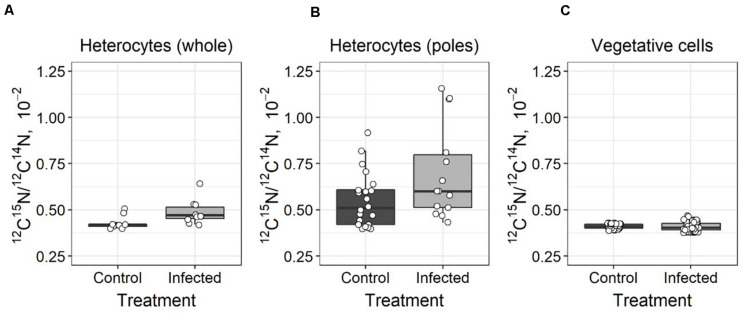
Differences in ^15^N enrichment in *Aphanizomenon flos-aquae* strain 2012/KM1/D3 heterocytes (whole cell–**A**, poles–**B**) and vegetative cells **(C)** indicated as ^12^C^15^N/^12^C^14^N ratio (calculated from nanoSIMS analysis). The solid line within the box marks the mean, the boundaries of the box represent the standard error, and the whiskers above and below the box show the standard deviation from the mean. White circles represent the data value of each measured heterocyte **(A,B)** or vegetative cell **(C)**.

**FIGURE 4:**
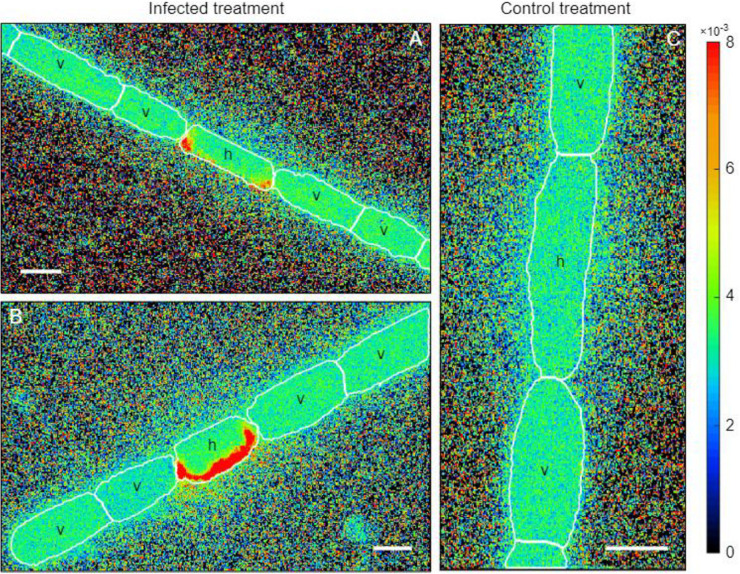
Nanometer-scale secondary ion mass spectrometry (nanoSIMS) micrographs of *Aphanizomenon flos-aquae* strain 2012/KM1/D3 heterocytes (h) and adjacent vegetative cells (v) in the infected **(A,B)** and control **(C)** treatments. The cellular nitrogen distribution is indicated as ^12^C^15^N/^12^C^14^N ratio.

The ^15^N enrichment was assessed using nanoSIMS analysis in both *A*. *flos-aquae* heterocytes and vegetative cells. Significantly increased (*t*-test, *t* = 6.92, *p* = 0.01, *df* = 19) ^15^N enrichment was detected in the heterocytes of the infected *A*. *flos-aquae* culture (Figures 3A, 4A), indicating accumulation of the newly fixed N_2_ within those cells. The local ^15^N enrichment at the poles of the heterocytes of the infected *A*. *flos-aquae* filaments were also significantly higher (*t*-test, *t* = 5.08, *p* = 0.03, *df* = 34) compared to the control cultures (Figures 3B, 4B). No statistically significant differences (*t*-test, *t* = 0.22, *p* = 0.82, *df* = 98) in whole-cell ^15^N enrichment in vegetative *A*. *flos*-*aquae* cells were found between the two treatments (Figure 3C), although the variation width in the infected cells was higher compared to the control. The nanoSIMS data showed that significant local ^15^N enrichments in vegetative cells were distributed randomly and occurred at a very low abundance (data not shown), having no impact on the average cell isotopic signal.

### Changes in Nutrient Concentration

**FIGURE 5:**
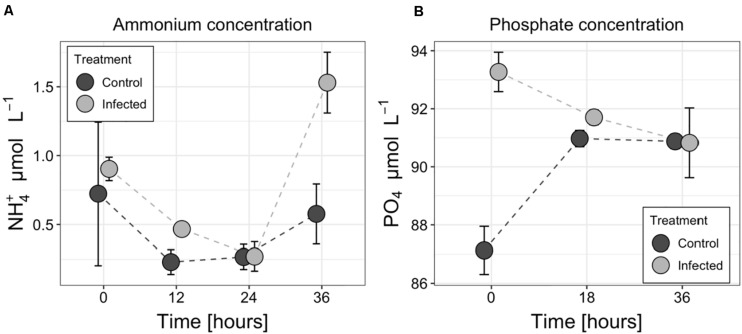
Changes in ammonium **(A)** and phosphate **(B)** concentrations in the medium of the control and infected cultures of the *Aphanizomenon flos-aquae* strain 2012/KM1/D3. Symbols indicate the mean values, and the error bar shows the standard deviation of three technical replicates.

Statistically significant (RM ANOVA *F* = 28.42, *p* = 0.01, *df* = 1) increase in ammonium concentration was observed in the medium of the infected culture compared to non-infected controls at 36 h (Figure 5A). The concentration of ammonium in the infected treatment changed from 0.90 μmol L^–1^ (±0.09 μmol L^–1^) at the beginning to 1.53 μmol L^–1^ (±0.22 μmol L^–1^) at the end of the experiment whereas in the controls it decreased from 0.72 μmol L^–1^ (±0.52 μmol L^–1^) to 0.58 μmol L^–1^ (±0.22 μmol L^–1^). Both curves had minimum concentrations between 12 and 24 h. The concentration of other inorganic nitrogen forms (nitrate and nitrite) remained below or just above the detection limit (0.2 μmol L^–1^ for nitrate, 0.05 μmol L^–1^ for nitrite) during the experiment and did not show any clear pattern (data not shown). The measured concentration of phosphates varied from 88.0 to 91.2 μmol L^–1^ and from 89.5 to 93.8 μmol L^–1^ in control and infected treatments, respectively (Figure 5B).

## Discussion

Understanding the factors controlling biological N_2_ fixation is important to fully comprehend the flow of energy and matter in the ecosystem. In this study, we analyzed the effect of cyanophage infection on nitrogen fixation and flow in diazotrophic cyanobacterium *A. flos-aquae*. We demonstrated that viral infection and diazotrophic activity in these filamentous cyanobacteria do not interfere with one another, indicating low degree of virus-mediated metabolic remodeling of *A*. *flos-aquae* heterocytes. Our results also suggest that CL 131 cyanophages contribute to nitrogen dynamics by (i) inducing alterations in nitrogen distribution within the infected *A*. *flos-aquae* filaments and (ii) through ammonium release by lysis.

The infection cycle of CL 131 progressed similarly to previously observed infection dynamics for this cyanophage (Šulčius et al., 2015a) as well as to that described for other cyanophages infecting filamentous cyanobacteria (Wang and Chen, 2008; Gao et al., 2009; Dwivedi et al., 2013; Watkins et al., 2014; Coloma et al., 2017; Šulčius et al., 2018). A significant increase in CL 131 abundance was detected 24–30 h after cyanophage addition (Figure 1), indicating lysis of *A*. *flos-aquae* cells and completion of the first infection cycle (Šulčius et al., 2015a). Changes in CL 131 abundance from T0 to T6 (Figure 1) imply slow CL 131 adsorption as only approximately 27% of the cyanophages were found associated with *A*. *flos-aquae* cells. Nevertheless, the calculated ratio of the adsorbed phages to each cyanobacterial cell was ∼1.7, suggesting that most of the cells were still infected. However, due to unsynchronized cell growth and division rates in filamentous cyanobacteria (Sakr et al., 2006), which likely occurs as a result of variation in metabolic and physiological state of each individual cell within the filaments, the increased variation in the latent period of the cyanophages and therefore differences in lysis timing may occur (Parada et al., 2006; Hyman and Abedon, 2009). Although both relatively low adsorption rates and unsynchronized cell lysis were also found for other virus and filamentous cyanobacteria host systems, eventually (e.g., after prolonged period of time and several infection cycles) most of the cells are lysed (Hewson et al., 2004; Gao et al., 2009; Šulčius et al., 2015a, 2018; Coloma et al., 2017). This further suggests that under conditions of massive infection and lysis such as for example during the blooms of filamentous cyanobacteria (Gons et al., 2002), a substantial amount of fixed N_2_ might be redirected toward the microbial loop.

### Nitrogen Fixation During Cyanophage Infection

To meet their N demand required for the efficient replication of viral nucleic acids and proteins within the infected cells, viruses are known to employ at least three different mechanisms for N acquisition: recycling of intracellular N sources (Ankrah et al., 2014), acquisition of extracellular nitrogen using host cell nitrogen uptake machinery (Waldbauer et al., 2019) and expression of host-derived viral genes involved in nitrogen metabolism (Yoshida et al., 2008; Monier et al., 2017). Noting that cyanophage CL 131 do not encode any known proteins involved in N metabolism (e.g., phycobilisomes degradation enzymes or N transporters; Šulčius et al., 2019), our findings suggest that cyanophage CL 131 replication strongly relies on the pre-existing intracellular nitrogen pool in *A*. *flos*-*aquae* cells. The major ecological implication, therefore, is that production rate (including latent period and burst size) of cyanophage CL 131 is more likely to be limited by the physiological state of *A*. *flos*-*aquae* and the ability of cyanophage to efficiently catabolize host intracellular compounds rather than by virus-mediated metabolic reprogramming of the host cells. This observation represents a relatively less common mode of the phage-host interactions that differ from other laboratory virus-host systems, including marine cyanobacteria, algae, and heterotrophic bacteria, in which viral replication significantly interfere with carbon and nitrogen cycling within the infected cells (reviewed in Zimmerman et al., 2020). Negligible effect of cyanophage infection on host metabolic activity was also observed for freshwater bloom-forming cyanobacteria *Microcystis aeruginosa* (Morimoto et al., 2018). The authors hypothesized that the lack of significant metabolic reprogramming of the infected cells might be associated with cyanophage avoidance strategy to prevent the activation of host defense systems, while material necessary for cyanophage DNA replication and protein synthesis can be derived from the host nucleotide and amino acid precursor pools (Morimoto et al., 2018).

The spatial separation of nitrogen fixation in heterocytes and cyanophage replication within the vegetative cells might explain the lack of interference between these two processes. Although N_2_ fixation and growth are usually tightly connected under relatively stable growth conditions (Muñoz-García and Ares, 2016), uncoupling between nitrogen and carbon metabolism may occur under environmental stress. Since heterocytes are capable of independent production of the reducing equivalents and ATP for the nitrogenase reactions in photosystem I (Magnuson and Cardona, 2016), nitrogen fixation can be maintained when growth conditions change. Thus, from the perspective of virus-mediated metabolic reprogramming of the infected cell, a process which essentially is a redirection of the energy supply from cellular reactions to the synthesis of viral particles (Puxty et al., 2015; Rosenwasser et al., 2016), the energy demanding process of N_2_ fixation taking place in physically separated and relatively energetically independent heterocyte, should not influence or restrict virus replication. This is because the required energy for virus replication within the vegetative cells is provided by other reactions such as for example pentose phosphate pathway (Thompson et al., 2011). Thus, such energetic “independence” of heterocytes and/or inability of a virus to access the ATP produced within the heterocytes makes possible that diazotrophic activity of the infected filaments is maintained, especially over the relatively short period of time, such as the time needed for cyanophage replication and assembly. Similar to our observations, studies with zooplankton predation (including *Daphnia*, *Diaptomus*, and *Bosmina*), which is another major biotic factor of cyanobacteria mortality, indicated that grazing on the vegetative cells of the filamentous cyanobacteria do not exert any effect on heterocyte activity and rates of nitrogen fixation (Shtina and Nekrasova, 1971; Chan et al., 2004), suggesting that heterocytes remain metabolically active even when vegetative cells are dying.

The importance of diazotrophically fixed nitrogen in the aquatic food web is strongly associated with its transformation within the cell (e.g., cellular stoichiometry and nutritional value) and a subsequent release into environment (e.g., in either dissolved or particulate form). In case of active virus-mediated metabolic reprogramming, as studies with marine heterotrophic bacteria and algae would suggest (reviewed in Zimmerman et al., 2020), the intracellular nitrogen resources should be effectively redirected from the production of cellular compounds toward new phage particles resulting in significant changes in cellular stoichiometry (nutritional value of the cell is changed; Ankrah et al., 2014; Monier et al., 2017). Alternatively, however, the metabolism of the assimilated nitrogen, including both synthesis and degradation of host proteins and various metabolites, might remain significantly unchanged during cyanophage infection (Morimoto et al., 2018), in turn, possibly having no impact on intracellular nutrient ratios (nutritional value of the cell does not change). Our observations, however, suggest another rather subtle effect of cyanophages on the N flow, when newly fixed nitrogen is neither incorporated into viral particles nor it accumulates into cyanobacteria biomass. Instead it remains in the heterocytes, changing N flow within the infected filaments and delaying any of its further transformations.

### Re-distribution of Fixed Nitrogen Within the Infected Filaments

In diazotrophic cyanobacteria, newly fixed N_2_ is either (i) transferred to the vegetative cells where it is used for cellular growth (biomass accumulation), (ii) released as ammonium (cellular exudation) to the surrounding environment, or (iii) under unfavorable growth conditions is converted into N storage compounds. These processes and thus the flow of nitrogen is actively regulated in cyanobacteria to maintain the beneficial interactions with other microorganisms and in response to environmental conditions to ensure cellular growth and survival (Flores and Herrero, 2005; Foster et al., 2011; Zehr and Kudela, 2011). Viral infection of the host cells has the potential to modulate the nitrogen metabolism and alter the flow of N within the infected cells (Brussaard et al., 2008; Ankrah et al., 2014). Both growth inhibition (Figure 1) and the lack of changes in N_2_ fixation activity (Figure 2) in the infected *A*. *flos*-*aquae* might suggest that newly fixed nitrogen is used for the production of cyanophage progeny rather than for incorporation into cyanobacterial biomass. In this case, and assuming that viruses are enriched in N compared to their hosts (Jover et al., 2014; Pasulka et al., 2018), increased local ^15^N enrichments relative to the control *A*. *flos-aquae* filaments can be expected in the cytoplasmic regions where the assembly of the newly produced cyanophage virions within the infected vegetative cell occurs. These regions can be clearly seen in the thin sections of the infected *A*. *flos*-*aquae* cells (for details see Šulčius et al., 2015a). Recent study has demonstrated the ability of nanoSIMS to resolve ^15^N enrichments in each individual viral particle (Pasulka et al., 2018). Thus, to test our hypothesis, we analyzed cellular distribution of ^15^N enrichments in vegetative cells of the infected and control cultures. The nanoSIMS analysis revealed no differences in both local (data not shown) and total (Figure 3C) ^15^N enrichments between infected and control vegetative *A*. *flos-aquae* cells, suggesting that only limited (if any) amount of newly fixed N_2_ is incorporated into cyanophage progeny. Also, this suggest that cyanophage infection does not result in the relative accumulation of nitrogen within infected cells as it was shown for some marine bacteria and algae virus-host systems (Ankrah et al., 2014; Monier et al., 2017).

In this study, we found different ^15^N enrichments in the heterocytes of the infected cultures compared to the uninfected controls (Figure 3A). The observed ^15^N enrichment in the heterocytes was also localized at its poles (at both ends close to the heterocyte neck; Figure 3B). The reason for this localization is not fully understood at this time but one possible explanation is nitrogen conversion into N storage compounds. It has been shown that heterocyte ends are the sites of accumulation of cyanophycin (Sherman et al., 2000) a polymer composed of aspartate and arginine that serves as a nitrogen reservoir (Flores et al., 2019). Cyanophycin granules were previously detected in the heterocytes of the field isolates of *A*. *flos-aquae*, yet at the relatively low concentrations, suggesting that fixed N was rapidly transported to the adjacent cells during population growth (Ploug et al., 2010). However, increased production of cyanophycin may occur under stress or growth-limiting conditions (Obst and Steinbüchel, 2006), including during the inhibition of host protein synthesis that is known to occur during viral infection (Rosenwasser et al., 2016). The accumulation of fixed N_2_ in the form of cyanophycin would be consistent with the observed ^15^N enrichments at heterocyte poles (Figure 3B) and growth inhibition of vegetative cells of *A*. *flos-aquae* by cyanophage CL 131 infection in our experiment (Figure 1). Consequently, it may further suggest that GS/GOGAT pathway, linking carbon and nitrogen metabolism in the filaments, may also be affected by the cyanophage infection. For example, one could expect that incorporation of fixed nitrogen into aspartate and arginine would also reduce the formation and transport of 2-oxoglutarate (2OG) and glutamine (Gln) into the vegetative cells. This in turn might result in downregulation of *NtcA* genes, global nitrogen regulators essential for regulation of the expression of numerous other important genes involved in maintenance of *A*. *flos-aquae* metabolism and physiology. Whether the above discussed phenomenon (cyanophycin accumulation in heterocytes and reduced supply of Gln and 2OG to vegetative cells) represents a case of coordinated cellular response to cyanophage infection remains unknown and further studies are needed to test this hypothesis. Nevertheless, the ecological implications could be that diazotrophically fixed nitrogen is neither accumulated into cyanobacteria biomass nor it is incorporated into newly synthesized phage particles. The nanoSIMS results suggest that the transport of newly fixed N_2_ from the heterocytes to the infected vegetative cells is suspended. The subsequent transformations of N_2_ is delayed and newly fixed N_2_ may not be released.

### Nitrogen Release by Cyanophage Infection and Lysis

Biological nitrogen fixation and its subsequent release is one of the largest sources of nitrogen enabling microbial community to alleviate general summer nitrogen limitation in many aquatic ecosystems (Vahtera et al., 2007; Degerholm et al., 2008; Wannicke et al., 2013; Karlson et al., 2015). Diazotrophically derived nitrogen can be released and transferred to co-occurring microbes either through the exudation of dissolved nitrogen products from cyanobacterial cells (mostly in the form of ammonium; Adam et al., 2016), zooplankton grazing (e.g., sloppy feeding and excretion of N compounds within the fecal pellets; Hasegawa et al., 2001), decay of the senescent cells (e.g., at the end of the vegetation period; Engström-Öst et al., 2013), viral lysis (Coloma et al., 2017) or combination of these processes. Although the type of material released by all of these processes includes a spectrum of compounds ranging from highly labile to recalcitrant, the stoichiometric composition and bioavailability of the released material is thought to be different (Suttle, 2007). It is generally believed that cellular exudation and lysis-mediated release of organic and inorganic molecules is more readily accessible to pelagic bacteria compared to zooplankton fecal pellets or decaying cells (Adam et al., 2016). Therefore, our observations revealing threefold higher ammonium concentration in the cell-free filtrates of the infected *A*. *flos-aquae* culture at the end of the incubation experiment (Figure 5A), would, therefore, tentatively suggest that cyanophage lysis can not only redirect the flow of nitrogen from cellular biomass (which otherwise would be grazed or decayed) toward higher N release, but also significantly increase N bioavailability in the system. It is worth to note that since the flow of newly fixed nitrogen is significantly reduced or inhibited in the infected filaments (as discussed above; Figures 3, 4), the released nitrogen should have probably been fixed and transferred to vegetative cyanobacterial cell before the cyanophage infection. Nevertheless, ammonium release would enhance N remineralization by uninfected bacteria and algae, having a net stimulatory effect on the growth of co-occurring microbial assemblages as it was experimentally shown for filamentous diazotrophic cyanobacterium *Nodularia spumigena* (Cairns et al., 2016) and natural phytoplankton community (Shelford et al., 2012). The extent of this effect, however, would depend on the host species (in our case *A*. *flos-aquae*), composition of the co-occurring community and the ability of each individual microbial species to assimilate lysis-released compounds.

On the other hand, it is also possible that infection and lysis of the vegetative cells would reduce or even prevent the supply of glutamate produced by GOGAT (glutamine oxoglutarate aminotransferase) in vegetative cells, which is used as a substrate by the glutamine synthetase (GS) in the heterocytes to produce Gln, a process during which fixed nitrogen is incorporated into amino acids. Therefore, one could hypothesize that ammonium produced in the un-infected and metabolically active heterocytes, and no longer being transferred (as Gln) to the adjacent lysed vegetative cells, is released into surrounding environment. Thus, future studies should include measurements of the GS/GOGAT activity to better understand nitrogen release from heterocytes during and after cyanophage infection.

Finally, no accompanied changes in phosphate concentration was found between the control and infected treatments at the lysis onset (36 h; Figure 5B), suggesting that viral lysis in addition to the induced alterations in the ammonium concentration (Figure 5A) and possibly in the prevailing N form in the environment, also have implications for the overall nutrient stoichiometry in aquatic ecosystems (Suttle, 2007). The observed differences in phosphate concentration between the infected and control cultures at T0 (Figure 5B) cannot be explained by any differences in the experimental setup, but would, however, have no substantial effect neither on *A*. *flos-aquae* growth nor for cyanophage infection process during the period of incubation experiment as the total P concentration far exceeded (>3-fold) the limit proposed to inhibit *A*. *flos-aquae* development (De Nobel et al., 1997; Takano and Hino, 2000; Vahtera et al., 2007).

## Conclusion

Determining the fate of diazotrophically derived nitrogen in the infected cells is important not only for the better assessment of the role of viruses in the nutrient flows and functioning of the microbial food web, but also for the development of more efficient ecosystem management plans. In this study, we described the effect of cyanophage infection on diazotrophic activity and N flow in the ecologically relevant heterocytous cyanobacterium *A. flos-aquae*. Although this represents a single case based on using the same initial culture and therefore may obscure the inference about the variance of the observed effect of infection in natural *A*. *flos-aquae* population consisting of various genotypes with different sensitivity to cyanophages, the random biological variation of *A*. *flos-aquae* filaments and individual cells, emerging due to its multicellular nature, asymmetric division and unsynchronized growth in our incubations might well reflect the general patterns of *A*. *flos-aquae* physiological state and fitness in aquatic environments, at least for the fraction of infection sensitive sub-population. Nevertheless, we demonstrated that infection with the cyanophage CL 131 does not inhibit the expression of *nifH* genes or the rate of net N_2_ assimilation (Figure 2); thus, the metabolism and activity of heterocytes remained unchanged. However, these observations may hinder the actual effect of viral infections on nitrogen dynamics and metabolism within host cells. The increased total (whole heterocytes; Figure 3A) and local (at the heterocyte poles; Figure 3B) ^15^N enrichment in the heterocytes of the infected *A*. *flos-aquae* culture indicates that the flow of fixed N_2_ within the filament might be altered as a result of viral infection. Whether and how these changes influence the stoichiometry and amino acid profiles of the infected vegetative cells of *A*. *flos-aquae* or, for example, the recovery of the resistant *A*. *flos-aquae* subpopulation remain to be elucidated. Finally, nitrogen that has been fixed before the infection of vegetative cyanobacterial cells takes place is released as ammonium (Figure 5A) or dissolved organic nitrogen upon cell lysis and would probably also change the flow of fixed N_2_ within the microbial food web (Shelford et al., 2012). We, thus, suggest that cyanophage infection redirect the pathway of fixed N_2_ from *A*. *flos-aquae* biomass (Figure 1) toward production of N storage compounds (Figure 3B) and enhanced N release (Figure 5A).

## Materials and Methods

### Experimental Organisms and Culture Conditions

The clonal cyanobacterium *A. flos-aquae* strain 2012/KM1/D3 was isolated from surface water collected from the Curonian Lagoon (N 55°30′, E 21°15′) in the south-eastern part of the Baltic Sea (Šulčius et al., 2015a) Unialgal yet non-axenic *A*. *flos-aquae* culture was grown in modified (pH 7.8–8.0) AF-6 medium (Watanabe et al., 2000) without the addition of a nitrogen source (AF-6N_0_) under a 14/10-h light-dark cycle with a light intensity of 30 μmol m^–2^ s^–1^ and at a constant 20°C temperature.

Cyanophage vB_AphaS-CL131 was isolated from the Curonian Lagoon (Lithuania) using *A*. *flos-aquae* strain 2012/KM1/D3 as a host organism (Šulčius et al., 2015b). CL 131 is a large (capsid size of ∼97 nm, tail length – ∼361 nm, genome size – ∼113 kb) virulent siphovirus with an infection cycle of ∼36 h (Šulčius et al., 2015b, 2019). Prior the experiment, CL 131 was propagated using exponential growth phase *A*. *flos-aquae* culture under the same conditions as those described above. Cyanophage inoculum for incubation experiment was prepared from *A*. *flos-aquae* lysates purified using cesium chloride (CsCl_2_) density gradient centrifugation (Šulčius et al., 2015b).

### Incubation Experiment and Sampling Scheme

**FIGURE 6:**
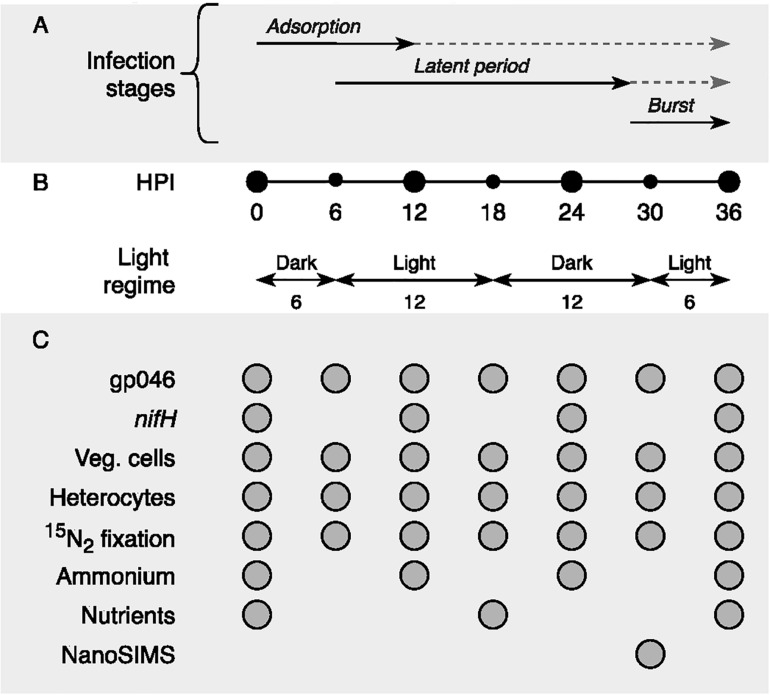
Schematic representation of **(A)** cyanophage vB_AphaS-CL131 infection stages (adsorption, latent period, and burst; modified from Šulčius et al., 2015a), **(B)** sampling points (black circles) in the context of light regime (hours), and **(C)** list of measurements taken during the course of the incubation experiment. HPI, hours post inoculation; Veg. cells, vegetative cells.

We limited our experiment to one infection cycle of the cyanophage CL 131 (Šulčius et al., 2015b) to emphasize the effect of cyanophage infection on N_2_ fixation and *nifH* gene expression in *A*. *flos-aquae* rather than the combined effect of infection and cell lysis. More specifically, we tried to prevent the potential effect of organic and inorganic nitrogen, which may be released into the medium due to cell lysis, on *A*. *flos-aquae* N metabolism. It has been suggested that these filamentous cyanobacteria may exhibit a preferential uptake of nitrates, nitrites, and ammonium instead of ^15^N_2_ (Layzell et al., 1985). The duration of the *A*. *flos-aquae* infection was determined in our previous studies and shown to be ∼36 h (Šulčius et al., 2015b). This period of time includes CL 131 adsorption, replication and burst (Šulčius et al., 2015b; Figure 6). In addition, the apparent growth rate of *A*. *flos-aquae* (0.24 d^–1^, corresponding to population doubling time of 2.89 days) was measured before the experiment to ensure that experiment will be performed within the same *A*. *flos-aquae* generation.

For incubation experiment, the early exponential growth phase cyanobacterial culture at the density of ∼2.5 × 10^5^ cells mL^–1^ was mixed with purified cyanophage CL 131 suspension at the density of ∼1.6 × 10^6^ phages mL^–1^, giving the ratio of phages added to each cyanobacterial cell of ∼6.5. The control treatment was amended with filter-sterilized AF-6N_0_ medium instead of cyanophage suspension. Both (infected and control) treatments were then subdivided into three technical replicates in glass septum bottles and were mixed by inverting them every 6 h. Nitrogen (^15^N_2_) gas (99.2 atom% ^15^N; Campro Scientific) was directly added as a bubble into incubation bottles (final concentration 0.2% v/v) of either infected or control cultures following descriptions in Montoya et al. (1996).

Samples for CL 131 and *A*. *flos-aquae* abundance and ^15^N_2_ fixation activity measurements were taken every 6 h. Samples for *nifH* gene expression and ammonium concentration measurements were taken every 12 h of the incubation experiment (Figure 6).

### RNA Extraction and cDNA Synthesis

Samples for RNA extraction were collected by filtering 24 mL of culture suspension onto Whatman polycarbonate filters (0.2 μm pore size, 47 mm diameter), flash frozen in liquid nitrogen and stored at −80°C until analysis. Total RNA was extracted following descriptions in Weinbauer et al. (2002) with modifications as in Glaubitz et al. (2009). The cell wall was mechanically disrupted using silanized glass beads (2–3 mm) in acidic extraction buffer [450 μL; 50 mM sodium acetate, 10 mM EDTA, 2% SDS (w/v), pH 4.2] mixed with equal volume of acidic phenol-chloroform (8:1, pH 4.2) suspension. Samples were homogenized and centrifuged for 5 min at 16,000 × g at 4°C. The aqueous phase containing RNA was transferred into new tube, washed twice with chloroform-isoamyl alcohol (24:1) and precipitated for 30 min at −80°C with isopropanol containing 3 M sodium acetate (0.1 volume) and 1.3 μL glycogen. The precipitated RNA was pelleted by centrifugation (60 min) and purified by washing twice with pre-cooled ethanol (70%, v/v). RNA was recovered from dry pellets by elution in nuclease-free DEPC-treated water and treated with DNase I (Turbo DNA-Free kit, Thermo Fisher Scientific) to remove the remaining DNA.

RNA samples were checked for DNA contamination using PCR and universal bacterial 16S rDNA gene primers (Com1/Com2; Table 1). RNA concentration and purity was assessed spectrophotometrically using the 2100 Electrophoresis Bioanalyzer (Agilent Technologies) and RNA 6000 Pico Kit (Agilent Technologies) following manufacturer’s recommendations. RNA was reverse transcribed into complementary DNA (cDNA) using MultiScribe Reverse Transcriptase (Thermo Fisher Scientific) following the manufacturer’s protocol. A PCR control without a reverse transcriptase and with a reaction mixture lacking template RNA were used to test the specificity of cDNA synthesis.

**TABLE 1:** Primer sets used for droplet digital PCR analysis of *nifH* gene expression (*nifH*), DNA contamination in RNA samples (16S) and cyanophage CL131 abundance (gp046).

Target gene	Forward primer (5′ to 3′)	Reverse primer (5′ to 3′)	Product length, bp	References
nifH	GATAGCTTTCTACGGCAAGGGC	GGTAGAGTCAGCTTTAGGATCG	124	This study
16S	GTGCCAGCMGCCGCGGTAA	GGACTACHVGGGTWTCTAAT	255	Caporaso et al., 2011
gp046	CACAACTCATTGCTGGTGGC	GGTTGGTGGTTCATAGGGCT	143	This study

### Primer Design and Validation

Specific primer pairs (Table 1) targeting *A*. *flos-aquae nifH* gene (Accession No. NZ_JSDP01000132.1) were designed with Primer3web software (v4.1.0; Untergasser et al., 2012). Primers specificity to ensure that they amplified only the target genetic locus were tested via conventional PCR using *A*. *flos-aquae* DNA (data not shown).

The CL 131-specific primers (Table 1) targeting so-far unique cyanophage structural gene (gp046; Accession No. ATW59314.1; Šulčius et al., 2019) were designed to give 143 bp amplicon with Primer3web software (v4.1.0; Untergasser et al., 2012) and tested for specificity with conventional PCR using cyanophage DNA and metagenomic analysis (for more details see Šulčius et al., 2019).

### Droplet Digital PCR

Droplet digital PCR (ddPCR) was performed using QX200^TM^ Droplet Digital PCR System (Bio-Rad Laboratories, Inc., Hercules, CA, United States). ddPCR reaction mixtures were prepared following manufacturer’s protocol. Each reaction mixture contained 1 ng of cDNA or 1 μL of particle-free filtrate (for CL 131 gp046 analysis). DEPC water and *A. flos-aquae* DNA was used instead of cDNA or particle-free filtrate as negative and positive controls, respectively. ddPCR reaction mixtures were loaded into Bio-Rad 8-chanell disposable droplet generator cartridge containing 70 μL of droplet generation oil in each well. Droplets were generated using QX200 droplet generator, transferred into a 96-well PCR plate, heat-sealed with a foil seal and amplified in a two (for *nifH*) or three-step (for *gp046*) PCR using conventional thermal cycler. PCR cycling conditions included an initial 5 min denaturing step at 95°C followed by 40 cycles of denaturation at 98°C for 30 s, annealing and elongation at 60°C for 60 s (for nifH) or 60°C for 90 s (for gp046), and a final signal stabilization step at 90°C for 5 min. After PCR, droplets were counted using QX200 droplet reader and analyzed with QuantaSoft software. Positive droplets, containing amplification products, were discriminated from negative droplets by setting the fluorescence threshold at the lowest point of the positive control droplet cluster and at the highest point of the negative control droplet cluster. The *nifH* transcript abundance was normalized for heterocyte abundance by dividing the number of *nifH* transcripts by the number of heterocytes recorded at each sampling point.

### Measurements of Nitrogen Fixation Rates

At each sampling point (Figure 6), 24 mL of culture suspension was filtered onto pre-combusted (450°C for 4.5 h) Whatman GF/F filters and stored at −20°C until further processing. Prior the measurements GF/F filters were dried at 60°C overnight and pelletized into tin cups, which then were analyzed with Thermo Flash 2000 elemental analyzer coupled to an DeltaV isotopic ratio mass spectrometer (Thermo Fisher Scientific). The rates of nitrogen fixation were calculated following descriptions given in Montoya et al. (1996) and normalized for heterocyte abundance.

### Microscopy Analysis

Samples (1 mL) for determination of *A*. *flos-aquae* cell density were preserved with formalin solution (final concentration of ∼1%) and kept in the dark at +4°C until further analysis. Vegetative cells and heterocytes of *A*. *flos-aquae* were enumerated at each sampling point under a light microscope (Nikon Eclipse C*_*i*_* H550S) using the Nageotte counting chamber and examining no less than 600 units. The *A*. *flos-aquae* population growth rates were calculated by fitting linear regressions to the natural log of cell abundance versus incubation time and calculating the regression slope.

### Nutrient Analysis

Samples (15 mL) for the analysis of dissolved inorganic nutrients [ammonium, nitrates and nitrites (NO_*X*_) and phosphates] were pre-filtered through Whatman PC filters (0.2 μm pore diameter) and measured spectrophotometrically according to Hansen and Koroleff (1999) using an automated constant flow analyzer Seal Analytical QuAAtro (for nitrates, nitrites and phosphates) or spectrophotometer Shimadzu, UV-1202 UV-Vis (for ammonium) with the detection limits of 0.2 μM L^–1^ for nitrate, 0.05 μM L^–1^ for nitrite, 0.1 μM L^–1^ for phosphate and 0.05 μM L^–1^ for ammonium.

### Nanoscale Secondary Ion Mass Spectrometry (NanoSIMS) Analysis

Subsamples (120 mL) for nanoSIMS analysis were taken at the lysis onset (30 h; Figure 6) and were additionally supplemented with 1 mL of ^15^N_2_ gas (99.2 atom% ^15^N; Campro Scientific) to increase net nitrogen dissolution to compensate for short incubation time. Subsamples were incubated for 6 h under the same conditions as described above and fixed with 37% formaldehyde (final concentration 2%). Culture suspension was serially diluted and filtered onto 0.2 μm Millipore polycarbonate filters (Billerica, MA, United States). The samples were coated with ca. 30 nm gold with a Cressington 108auto sputter coater (Watford, United Kingdom). SIMS imaging was performed using a NanoSIMS 50 L instrument (Cameca, France) at the Leibniz-Institute for Baltic Sea Research Warnemünde (IOW). A ^133^Cs^+^ primary ion beam was used to erode and ionize atoms of the sample. Among the received secondary ions, images of ^12^C^–^, ^13^C^–^, ^12^C^14^N^–^, ^12^C^15^N^–^ were recorded simultaneously for areas of interest using mass detectors equipped with electron multipliers (Hamamatsu). The mass resolving power was adjusted to be sufficient to suppress interferences at all masses allowing, e.g., the separation of ^13^C^–^ from interfering ions such as ^12^C^1^H^–^. Prior to the analysis, sample areas of 40 × 40 μm were sputtered for 4:30 min with 600 pA to erode the gold, clean the surface, reach the steady state of secondary ion formation and penetrate into the cell. The primary ion beam current during the analysis was 3 pA; the scanning parameters were 512 × 512 pixels for areas of 35 × 35 μm, with a dwell time of 250 μs per pixel. Sixty planes were analyzed.

Samples were analyzed with the Look@NanoSIMS software (Gregor and Maršálek, 2004). The planes were checked for inconsistencies, drift corrected based on the ^12^C^14^N^–^ signals and accumulated. Regions of interest (ROIs) for the calculation of ratios were drawn according to the cell outlines of the ^12^C^14^N^–^ signals. The ROI outlines were crosschecked and corrected with the cell appearances of ^12^C^15^N^–^ signals to include the areas with high enrichments. In parallel ROIs on the filter surface were defined for quality control of the measurement and correction of instrumental offset. For each ROI a ^12^C^15^N^–^/^12^C^14^N^–^ ratio was calculated. The ^12^C^15^N^–^/^12^C^14^N^–^ ratio of the averaged filter areas was used to calculate a correction value to reach nominal values (0.0036734) for the areas outside the cells. The cell values were corrected with this value. Thus, if enrichment material, e.g., from lysed cells, settled on the filter, the received values might underestimate the activity of the cells.

For two randomly selected spots additional implantation and analysis was performed to penetrate deeper in the cell. The results did not change in deeper cell parts but we must admit, that no cell was consumed in total by the analysis. Parallel to ^12^C^15^N^–^/^12^C^14^N^–^ ratios also the ^13^C^–^/^12^C^–^ ratios were analyzed routinely. No enrichments occurred there. This points to the fact, that the observed enrichments are no methodical artifact by e.g., topography.

### Statistical Analysis

Differences in *nifH* transcript abundance and nitrogen fixation rates were compared between treatments over the time course of the experiment using a repeated-measures ANOVA (RM-ANOVA). Variables were tested for normality and homogeneity of variance using the Shapiro–Wilk and Levene’s test, respectively, and log10-transformed if needed to meet the assumption of normality. Additionally, independent samples t-test assuming equal variances was applied to find out whether ^15^N enrichments, *A*. *flos-aquae* growth rate and nutrient concentrations differed from the control or from each other on specific sampling points (both within and between the treatments). All statistical analyses were performed using RStudio (v. 1.2.5001) with R version 3.6.1, and with a *p*-value < 0.05 considered as significant.

## Data Availability Statement

The raw data supporting the conclusions of this article will be made available by the authors, without undue reservation.

## Author Contributions

SŠ and JK conceived the study and analyzed the data. JK performed the experiments, collected and processed the samples, and conducted laboratory measurements. AV performed nanoSIMS and analyzed the data. EŠ designed PCR primers for *nifH* gene in *A*. *flos-aquae*. SŠ wrote the manuscript. All authors contributed to manuscript editing.

## Conflict of Interest

The authors declare that the research was conducted in the absence of any commercial or financial relationships that could be construed as a potential conflict of interest.
